# Hepatitis E Virus in Rabbits, Virginia, USA

**DOI:** 10.3201/eid1711.110428

**Published:** 2011-11

**Authors:** Caitlin M. Cossaboom, Laura Córdoba, Barbara A. Dryman, Xiang-Jin Meng

**Affiliations:** Virginia Polytechnic Institute and State University, Blacksburg, Virginia, USA

**Keywords:** hepatitis E, hepatitis E virus, viruses, rabbits, genotype 3, zoonoses, zoonotic infection, animal reservoirs, Virginia, United States

## Abstract

We identified hepatitis E virus (HEV) in rabbits in Virginia, USA. HEV RNA was detected in 14 (16%) of 85 serum samples and 13 (15%) of 85 fecal samples. Antibodies against HEV were detected in 31 (36%) of 85 serum samples. Sequence analyses showed that HEV from rabbits is closely related to genotype 3.

Hepatitis E virus (HEV), the causative agent of hepatitis E, is a major human pathogen and a public health concern in many developing countries. Sporadic cases of acute hepatitis E have also been reported in many industrialized countries, including the United States (*1*). In addition to humans, strains of HEV have also been genetically identified from other animal species, including pigs, chickens, rats, mongooses, and deer (*2*). A unique strain of HEV was recently identified from farmed rabbits in the People’s Republic of China (*3**,**4*), although its prevalence in other regions is unknown.

At least 4 major genotypes of HEV that infect mammals have been identified (*5*). Genotypes 1 and 2 are restricted to humans, and genotypes 3 and 4 have an expanded host range and are capable of causing zoonotic disease (*6**–**10*). Avian HEV from chickens likely represents a new genus within the family *Hepeviridae* (*1**,**2*). More recently, additional putative new genotypes of HEV were identified in rats in Germany (*11*) and wild boars in Japan (*12*). The objectives of this study were to determine if farmed rabbits in the United States are infected by HEV, and if so, to genetically identify these viruses from rabbits in the United States.

## The Study

Fecal swab specimens and serum samples were obtained from 85 rabbits from 2 rabbit farms in Virginia, USA (25 rabbits from Farm A and 60 rabbits from Farm B). The 2 rabbit farms, 1 in eastern Virginia and 1 in southwestern Virginia, raised rabbits for meat consumption, fur, and pets. Ages of rabbits ranged from 3.9 to 36.8 months (mean 7.0 months) on Farm A and from 3.0 to 56.9 months (mean 10.8 months) on Farm B. Rabbits were of various breeds, including Californian, Flemish X, Lop, MiniRex, New Zealand, New Zealand X, Rex X, Salitan, and TN Redback. All rabbits appeared to be healthy.

Serum samples were tested for antibodies against HEV by using an ELISA essentially as described (*13*). A truncated recombinant genotype 1 HEV capsid protein containing the immunodominant 452–617 aa region (GenWay Biotech, Inc., San Diego, CA, USA) was used as antigen. Horseradish peroxidase–conjugated goat anti-rabbit IgG (Kirkegaard and Perry Laboratories, Gaithersburg, MD, USA) was used as the secondary antibody. Preinoculation serum and convalescent-phase serum obtained from 2 rabbits experimentally infected with rabbit HEV were included as negative and positive controls, respectively. The ELISA cutoff was calculated as the mean negative control optical density value plus 3 SD. The prevalence of IgG against HEV was 36.5% (31/85): 52% (13/25) for Farm A rabbits and 30% (18/60) for Farm B rabbits (Table 1).

**Table 1:** HEV in rabbits from 2 farms in Virginia, USA*

Farm	No. rabbits tested	Mean age, mo	No. (%) positive for antibodies against HEV	No. (%) positive for HEV RNA		No. (%) exposed to HEV
				Serum	Feces	
A	25	7.0	13 (52.0)	12 (48.0)	10 (40.0)	20 (80.0)
B	60	10.8	18 (30.0)	2 (3.3)	3 (5.0)	22 (36.7)
Total	85	9.7	31 (36.5)	14 (16.5)	13 (15.3)	42 (49.4)

*HEV, hepatitis E virus.

All rabbit serum and fecal swab samples were tested for HEV RNA by using a nested reverse transcription PCR and a set of degenerate primers that amplify a conserved region of the HEV capsid gene. These primers were designed on the basis of multiple sequence alignment of the 2 known rabbit strains of HEV from China (*3**,**4*) and 75 other genotype 3 HEV strains. Primers used for the first-round PCR were forward primer RabdegF1 (5′-GCMACACGKTTYATGAARGA-3′) and reverse primer RabdegR1 (5′-ACYTTRGACCAATCVAGRGARC-3′). Primers used for the second-round PCR were forward primer RabdegF2 (5′-GCTGAYACRCTTCTYGGYG-3′) and reverse primer RabdegR2 (5′-TGAMGGRGTRGGCYGRTCYTG-3′).

HEV RNA was detected in 19 (22%) of 85 rabbits, including 14 (16%) of 85 serum samples and 13 (15%) of 85 fecal samples (Table 1). Authenticity of amplified PCR products was confirmed by sequencing. More rabbits were positive for HEV RNA on Farm A (48% and 40% in serum and fecal samples, respectively) than on Farm B (3% and 5% in serum and fecal samples, respectively) (Table 1). A total of 42 (49%) of 85 rabbits on the 2 farms were infected (fecal shedding, viremia, or seropositive): 20 (80%) of 25 on Farm A and 22 (37%) of 60 on Farm B (Table 1).

A 181-bp sequence within the capsid gene was identified in all 27 PCR-positive samples amplified. Sequence analyses identified 4 HEV isolates: USRab-14, USRab-16, USRab-31, and USRab-52. The 4 rabbit HEV isolates shared ≈81.2%–97.8% nt sequence identity with each other and 80.1%–95.6% nt sequence identity with the 2 rabbit HEV isolates from China (GDC9 and GDC46) (*3*). Small amounts of available clinical samples limited our ability to perform extensive genetic characterization of rabbit HEV isolates.

However, we amplified a larger 765-bp sequence within the capsid gene of isolate USRab-14 (Figure) by using a set of heminested primers: first-round PCR with primers RabdegF2 and RabOrf2R1 (reverse 5′-TTAAAACTCCCGGGTTTTACC-3′) and second-round PCR with primers RabOrf2F2 (forward 5′-CAGGTATTCTACTCCCGC-3′) and RabOrf2R1. Analysis of the 765-bp sequence (GenBank accession no. JN383986) showed that the USRab-14 isolate shared ≈87%–89% nt sequence identity with the 2 rabbit HEV strains from China (Table 2). Phylogenetic analysis showed that the USRab-14 isolate grouped with the 2 rabbit HEV strains from China (GDC9 and GDC46) (Figure), which are more closely related to genotype 3 HEV than to any other known HEV genotypes (Figure; Table 2). These results suggest that the rabbit HEV is likely a distant member of genotype 3 (*14*).

**Figure:**
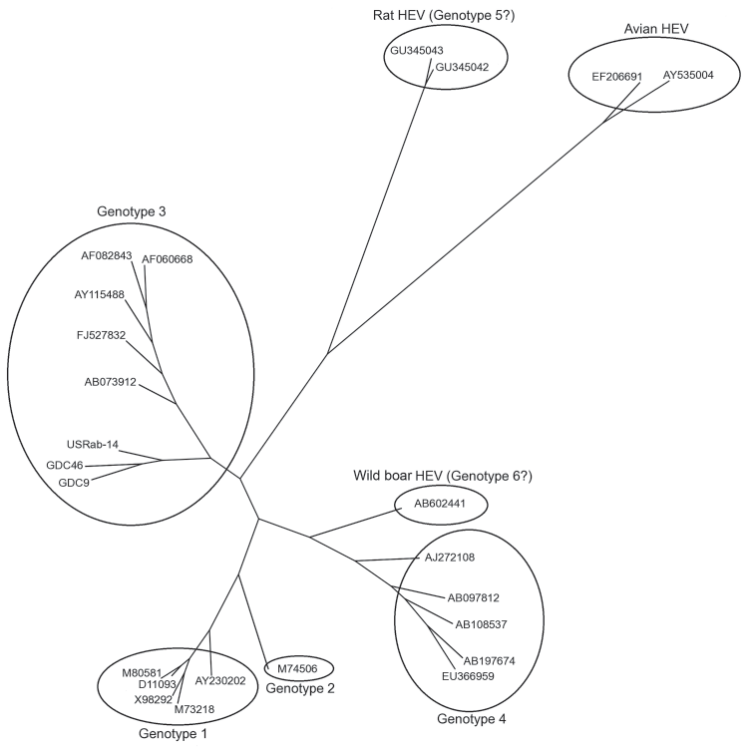
Phylogenetic tree for the 765-bp sequence of open reading frame 2 of the capsid gene of rabbit hepatitis E virus (HEV) isolate USRab-14 from the United States, 2 rabbit HEV isolates (GDC9 and GDC46) from China, representative genotype 1–4 HEV strains, avian HEV, rat HEV, and novel wild boar HEV. GenBank accession numbers are shown for each HEV strain used in the phylogenetic analysis.

**Table 2:** Nucleotide sequence identities of a 765-bp capsid gene sequence among HEV strains from rabbits in China and the United States and other HEV strains*

Virus isolate	HEV strains, % identity					
	China	Genotype 1	Genotype 2	Genotype 3	Genotype 4	Avian
USRab-14	87.2–89.0	78.3–79.3	74.8	80.1–82.3	78.9–81.0	34.9–35.9
China GDC9/GDC46	NA	77.2–78.6	74.5–75.5	78.7–83.1	78.0–82.0	35.3–36.0

*HEV, hepatitis E virus; NA, not applicable.

## Conclusions

We report that farmed rabbits in the United States are naturally infected with antibodies against HEV and that HEV RNA was detected in various breeds of rabbits from 2 farms in Virginia, USA. The prevalence of antibodies against HEV and HEV RNA was higher on Farm A than on Farm B. This variation may reflect differences in rabbit housing practices on the 2 farms: rabbits on Farm A were caged in groups of 2–9 and rabbits on Farm B were each caged individually. Because HEV is transmitted by the fecal–oral route, virus likely spreads between cage mates on Farm A, thus increasing the numbers of HEV-positive rabbits.

The overall prevalence of antibodies against HEV (36%) among rabbits from the United States was lower than that among rabbits from Gansu and Beijing, China (57% and 55%, respectively) (*3**,**4*). The prevalence of HEV RNA in serum and fecal samples on rabbit farms in the United States (16.5% and 15.3%, respectively) was higher than that on farms in Gansu and Beijing, China (7.5% and 6.96%, respectively). Ages of rabbits, animal housing practices, and hygienic conditions on rabbit farms may explain the observed difference in HEV prevalence.

Sequence analysis of the 765-bp capsid gene showed that HEV isolate USRab-14 from the United States is genetically different from the 2 rabbit strains of HEV from China (Table 2; Figure). Genetic variations were also observed among the 4 rabbit HEV isolates from the 2 rabbit farms in the United States. Rabbit HEV strains from the United States and China clustered into a distinct branch closely related to genotype 3 HEV.

Thus, similar to swine HEV in pigs (*13*) and avian HEV in chickens (*15*), rabbit HEV is also widespread in the rabbit population in the United States. The fact that rabbit HEV appears to be closely related to genotype 3 HEV raises a potential concern for zoonotic infection because genotype 3 HEV from other animal species is known to infect humans (*1**,**2*). Therefore, cross-species infection and zoonotic risk for infection with rabbit HEV should be evaluated.
